# Supplementary material for: Mineral Acid Co-Extraction in Reactive Extraction of Lactic Acid Using a Thymol-Menthol Deep Eutectic Solvent as a Green Modifier
Source: Molecules. 2024 Apr 11;29(8):1722. doi: 10.3390/molecules29081722 (PMC11052331; doi:10.3390/molecules29081722)
Supplement: Supplementary file 1 [file molecules-29-01722-s001.zip › molecules-2951464-supplementary.pdf]

Supplementary materials

Supplementary materials: Mineral acid co-extraction in reactive extraction of lactic acid using a thymol-menthol deep eutectic solvent as a green modifier

Paul Demmelmayer [1], Marija Ćosić [1] and Marlene Kienberger [1,*]

[1] Institute of Chemical Engineering and Environmental Technology, Graz University of Technology, Inffeldgasse 25/C 8010 Graz Austria; demmelmayer@tugraz.at; cosic.m@outlook.com.
* Correspondence: marlene.kienberger@tugraz.at

Mass balance for the extraction step

The overall mass balances for the extraction step is shown in Equation S1 with the volume (V) and acid concentration (c) for the feed (F), solvent (S), raffinate (R), and extract (E) phases.

$$V_F \cdot c_F + V_S \cdot c_S = V_R \cdot c_R + V_E \cdot c_E \tag{S1}$$

In the next step, Equation S1 is rearranged to Equation S2. Here, the volume of the raffinate phase is replaced by $V_R = V_F - \Delta V$ and the volume of the extract phase is substituted by $V_E = V_S + \Delta V$. The term ΔV accounts for the volume change due to water transport from the feed phase into the solvent phase. The volume change because of solvent phase loss into the aqueous phase and acid transport into the solvent phase is neglected.

$$c_E = (V_F \cdot c_F - (V_F - \Delta V) \cdot c_R)/(V_S + \Delta V) \tag{S2}$$

To calculate the volume change ΔV, Equation S3 is used using the mass of water contained in the extract phase ($m_{w,E}$), the mass of water contained in the solvent phase ($m_{w,S}$), and the density of water (ρ_w).

$$\Delta V = (m_{w,E} - m_{w,S})/\rho_w \tag{S3}$$

The $m_{w,S}$ is calculated according to Equation S4 using the total mass of the solvent phase (m_S) and the mass fraction of water in the solvent phase ($w_{w,S}$).

$$m_{w,S} = m_S \cdot w_{w,S} \tag{S4}$$

The $m_{w,E}$ is determined using Equation S5 with the total mass of the extract phase (m_E) and the mass fraction of water in the extract phase ($w_{w,E}$).

$$m_{w,E} = m_E \cdot w_{w,E} \tag{S5}$$

As weighing of the extract phase after the experiment results in errors due to the extract phase sticking to the separatory funnel walls, m_E is calculated according to Equation S6; $m_{inert,E}$ denotes the mass of the inert part of the extract phase (extract phase without water), and $w_{inert,E}$ denotes the mass fraction of the inert part of the extract phase.

$$m_E = m_{inert,E}/w_{inert,E} = (m_S - m_{w,S})/(1 - w_{w,E}) \tag{S6}$$

When compared to the simplified method of assuming a constant phase volumetric phase ratio of one, the presented way of evaluation tackles errors in calculations due to high water transport into to solvent phase, e.g. a change from 0 wt% water in the solvent phase to 10 wt% of water in the extract phase.

Table S1. The extractant loading (z_{acid}) for HCl, HNO_3, H_2SO_4, and H_3PO_4 for the reactive extraction from the single-acid model solutions using TOA, TOPO, or TBP diluted in 1-octanol, tmDES, or limonene at 25 ± 0.5 °C. The volumetric feed-to-solvent phase ratio was one.

**Extractant**	**Modifier/ diluent**	z_{HCl}	z_{HNO3}	z_{H2SO4}	z_{H3PO4}
TOA	1-Octanol	2.07 ± 0.00	2.01 ± 0.00	2.02 ± 0.00	2.09 ± 0.00
	tmDES	1.31 ± 0.01	1.27 ± 0.01	0.984 ± 0.001	0.658 ± 0.012
	Limonene	1.20 ± 0.01	1.17 ± 0.00	0.874 ± 0.006	0.624 ± 0.008
TOPO	1-Octanol	0.111 ± 0.003	0.160 ± 0.009	0.131 ± 0.015	0.253 ± 0.003
	tmDES	0.0455 ± 0.0103	0.0513 ± 0.0142	0.183 ± 0.004	0.238 ± 0.028
	Limonene	0.0514 ± 0.0022	0.171 ± 0.001	0.0707 ± 0.0100	0.247 ± 0.001
TBP	1-Octanol	0.0213 ± 0.0038	0.156 ± 0.021	0.0938 ± 0.0128	0.250 ± 0.027
	tmDES	0.163 ± 0.006	0.116 ± 0.001	0.159 ± 0.005	0.277 ± 0.018
	Limonene	0.122 ± 0.002	0.0923 ± 0.0094	0.167 ± 0.008	0.234 ± 0.001

Table S2. The extractant loading (z_{acid}) for HCl, HNO_3, H_2SO_4, and H_3PO_4 for the reactive extraction from the multi-acid model solution using TOA, TOPO, or TBP diluted in 1-octanol, tmDES, or limonene at 25 ± 0.5 °C. The volumetric feed-to-solvent phase ratio was one.

**Extractant**	**Modifier/ diluent**	z_{HCl}	z_{HNO3}	z_{H2SO4}	z_{H3PO4}
TOA	1-Octanol	0.337 ± 0.008	0.389 ± 0.009	0.268 ± 0.005	0.0782 ± 0.0027
	tmDES	0.329 ± 0.001	0.337 ± 0.001	0.190 ± 0.001	0.0776 ± 0.0002
	Limonene	0.277 ± 0.000	0.290 ± 0.000	0.147 ± 0.001	0.0692 ± 0.0077
TOPO	1-Octanol	0.0213 ± 0.0043	0.0262 ± 0.0031	0.0115 ± 0.0031	0.0190 ± 0.0029
	tmDES	0.0158 ± 0.0011	0.0214 ± 0.0031	0.0249 ± 0.0027	0.0218 ± 0.0027
	Limonene	0.0127 ± 0.0033	0.0105 ± 0.0033	0.0181 ± 0.0042	0.0183 ± 0.0045
TBP	1-Octanol	0.0235 ± 0.0007	0.0198 ± 0.0013	0.0154 ± 0.0020	0.0183 ± 0.0022
	tmDES	0.0224 ± 0.0016	0.0203 ± 0.0010	0.0176 ± 0.0004	0.0115 ± 0.0010
	Limonene	0.00586 ± 0.00187	0.0174 ± 0.0005	0.0173 ± 0.0012	0.00795 ± 0.00274

Table S3. The extractant loading for LA (z_{LA}) and the mineral acid ($z_{mineral}$) for the reactive extraction of LA from a model solution using TOA, TOPO, or TBP diluted in 1-octanol, tmDES, or limonene at 25 ± 0.5 °C. The model solutions contained LA and one mineral acid, and the volumetric feed-to-solvent phase ratio was one.

Mineral acid	Solvent phase	z_{LA}	$z_{mineral}$
HCl	TOA:1-octanol	0.292 ± 0.004	0.969 ± 0.002
	TOA:tmDES	0.167 ± 0.003	0.919 ± 0.011
	TOA:limonene	0.102 ± 0.002	0.878 ± 0.000
	TOPO:tmDES	0.0733 ± 0.0027	0.0215 ± 0.0005
HNO_3	TOA:1-octanol	0.199 ± 0.005	0.943 ± 0.002
	TOA:tmDES	0.129 ± 0.005	0.945 ± 0.000
	TOA:limonene	0.0909 ± 0.0047	0.916 ± 0.004
	TOPO:tmDES	0.0746 ± 0.0023	0.0235 ± 0.0007
H_2SO_4	TOA:1-octanol	0.212 ± 0.004	0.594 ± 0.004
	TOA:tmDES	0.130 ± 0.026	0.561 ± 0.005
	TOA:limonene	0.124 ± 0.002	0.549 ± 0.010
	TOPO:tmDES	0.0819 ± 0.0038	0.0260 ± 0.0000
H_3PO_4	TOA:1-octanol	0.455 ± 0.002	0.541 ± 0.009
	TOA:tmDES	0.441 ± 0.003	0.467 ± 0.003
	TOA:limonene	0.112 ± 0.009	0.184 ± 0.001
	TOPO:tmDES	0.0694 ± 0.0001	0.0238 ± 0.0011

Table S4. The LA concentration in the extract phase ($c_{LA,E}$) and mineral acid concentration in the extract ($c_{mineral,E}$) for back-extraction experiments. The extract phases were from experiments performed in section 2.4, the extraction temperature was 25 ± 0.5 °C, and the volumetric feed-to-solvent phase ratio was one.

Mineral acid	Solvent phase	$c_{LA,E}$ [mol·L^{-1}]	$c_{mineral,E}$ [mol·L^{-1}]
HCl	TOA:1-octanol	0.0585 ± 0.0013	0.194 ± 0.001
	TOA:tmDES	0.0334 ± 0.0010	0.184 ± 0.003
	TOA:limonene	0.0204 ± 0.0005	0.175 ± 0.000
	TOPO:tmDES	0.0147 ± 0.0008	0.00429 ± 0.00013
HNO_3	TOA:1-octanol	0.0398 ± 0.0014	0.189 ± 0.001
	TOA:tmDES	0.0258 ± 0.0015	0.189 ± 0.000
	TOA:limonene	0.0182 ± 0.0013	0.183 ± 0.001
	TOPO:tmDES	0.0149 ± 0.0007	0.00470 ± 0.00018
H_2SO_4	TOA:1-octanol	0.0423 ± 0.0011	0.119 ± 0.001
	TOA:tmDES	0.0260 ± 0.0073	0.112 ± 0.002
	TOA:limonene	0.0249 ± 0.0005	0.110 ± 0.003
	TOPO:tmDES	0.0164 ± 0.0011	0.00520 ± 0.00000
H_3PO_4	TOA:1-octanol	0.0912 ± 0.0006	0.108 ± 0.003
	TOA:tmDES	0.0884 ± 0.0010	0.0935 ± 0.0008
	TOA:limonene	0.0224 ± 0.0027	0.0367 ± 0.0004
	TOPO:tmDES	0.0139 ± 0.0000	0.00477 ± 0.00030
